# Supplementary material for: For 481 biomedical open access journals, articles are not searchable in the Directory of Open Access Journals nor in conventional biomedical databases
Source: PeerJ. 2015 May 19;3:e972. doi: 10.7717/peerj.972 (PMC4451041; doi:10.7717/peerj.972)
Supplement: Table S1 — Numbers in parentheses are the number of journals for each subject. Journals can have more than one subject. [file peerj-03-972-s002.docx]

| Subject term (no. of journals) | |
| --- | --- |
| Anesthesiology (22) | Medical physics. Medical radiology. Nuclear medicine (25) |
| Arctic medicine. Tropical medicine (11) | Medical technology (6) |
| Biochemistry (46) | Medicine (2266) |
| Biotechnology (57) | Medicine (General) (615) |
| Computer applications to medicine. Medical informatics (14) | Microbiology (64) |
| Cytology (15) | Neoplasms. Tumors. Oncology. Including cancer and carcinogens (89) |
| Dentistry (105) | Neurology. Diseases of the nervous system (136) |
| Dermatology (27) | Neurosciences. Biological psychiatry. Neuropsychiatry (195) |
| Diseases of the blood and blood-forming organs (27) | Nursing (53) |
| Diseases of the circulatory (Cardiovascular) system (82) | Nutritional diseases. Deficiency diseases (6) |
| Diseases of the digestive system. Gastroenterology (47) | Ophthalmology (37) |
| Diseases of the endocrine glands. Clinical endocrinology (27) | Orthopedic surgery (22) |
| Diseases of the genitourinary system. Urology (34) | Otorhinolaryngology (28) |
| Diseases of the musculoskeletal system (20) | Pathology (34) |
| Diseases of the respiratory system (22) | Pharmacy and materia medica (91) |
| Pediatrics (62) | Physiology (34) |
| Genetics (58) | Psychiatry (82) |
| Geriatrics (19) | Public aspects of medicine (231) |
| Gynecology and obstetrics (44) | Specialties of internal medicine (301) |
| Human anatomy (12) | Sports medicine (24) |
| Immunologic diseases. Allergy (38) | Surgery (131) |
| Industrial medicine. Industrial hygiene (3) | Therapeutics. Pharmacology (98) |
| Infectious and parasitic diseases (41) | Therapeutics. Psychotherapy (15) |
| Internal medicine (779) | Toxicology. Poisons (20) |
| Medical emergencies. Critical care. Intensive care. First aid (29) | Veterinary medicine (58) |
| Medical philosophy. Medical ethics (6) |  |
