# Supplementary material for: For 481 biomedical open access journals, articles are not searchable in the Directory of Open Access Journals nor in conventional biomedical databases
Source: PeerJ. 2015 May 19;3:e972. doi: 10.7717/peerj.972 (PMC4451041; doi:10.7717/peerj.972)
Supplement: Table S2 — All subsets were considered biomedical. [file peerj-03-972-s003.docx]

| SCOPUS biomedical subject terms (no. of subsets) |
| --- |
| Biochemistry, Genetics and Molecular Biology (15) |
| Immunology and Microbiology (6) |
| Medicine (48) |
| Neuroscience (9) |
| Nursing (23) |
| Pharmacology, Toxicology and Pharmaceutics (5) |
| Dentistry (6) |
| Veterinary (4) |
| Health Professions (16) |
| Total of 9 subjects (132) |
