# Supplementary material for: For 481 biomedical open access journals, articles are not searchable in the Directory of Open Access Journals nor in conventional biomedical databases
Source: PeerJ. 2015 May 19;3:e972. doi: 10.7717/peerj.972 (PMC4451041; doi:10.7717/peerj.972)
Supplement: Table S3 — ISSN; International Standard Serial Number, XML; eXtensible Markup Language. [file peerj-03-972-s004.docx]

| **XML term** | **Variable** |
| --- | --- |
| <dc:date> | Date published |
| <dc:identifier> | ISSN |
